# Developing Reporting Guidelines for Social Media Research (RESOME) by Using a Modified Delphi Method: Protocol for Guideline Development

**DOI:** 10.2196/31739

**Published:** 2022-05-09

**Authors:** Aradhna Kaushal, Caroline Bravo, Stephen Duffy, Douglas Lewins, Ralph Möhler, Rosalind Raine, Ivo Vlaev, Jo Waller, Christian von Wagner

**Affiliations:** 1 Research Department of Behavioural Science and Health University College London London United Kingdom; 2 Ontario Health (Cancer Care Ontario) Toronto, ON Canada; 3 Policy Research Unit in Cancer Awareness, Screening and Early Diagnosis Wolfson Institute of Population Health Queen Mary University of London London United Kingdom; 4 Institute for Health Services Research and Health Economics, Centre for Health and Society Faculty of Medicine Heinrich-Heine-University Düsseldorf Düsseldorf Germany; 5 Department of Applied Health Research University College London London United Kingdom; 6 Behavioural Science Group Warwick Business School University of Warwick Coventry United Kingdom; 7 School of Cancer and Pharmaceutical Sciences Faculty of Life Sciences and Medicine King’s College London London United Kingdom

**Keywords:** social media, research design, web-based social networking, health behavior, health promotion, public health

## Abstract

**Background:**

Social media platforms, such as Facebook, Twitter, and Instagram, are being increasingly used to deliver public health interventions. Despite the high level of research interest, there is no consensus or guidance on how to report on social media interventions. Reporting guidelines that incorporate elements from behavior change theories and social media engagement frameworks could foster more robust evaluations that capture outcomes that have an impact on behavior change and engagement.

**Objective:**

The aim of this project is to develop, publish, and promote a list of items for our Reporting Guidelines for Social Media Research (RESOME) checklist.

**Methods:**

RESOME will be developed by using a modified Delphi approach wherein 2 rounds of questionnaires will be sent to experts and stakeholders. The questionnaires will ask them to rate their agreement with a series of statements until a level of consensus is reached. This will be followed by a web-based consensus meeting to finalize the reporting guidelines. After the consensus meeting, the reporting guidelines will be published in the form of a paper outlining the need for the new guidelines and how the guidelines were developed, along with the finalized checklist for reporting. Prior to publication, the guidelines will be piloted to check for understanding and simplify the language used, if necessary.

**Results:**

The first draft of RESOME has been developed. Round 1 of the Delphi survey took place between July and December 2021. Round 2 is due to take place in February 2022, and the web-based consensus meeting will be scheduled for the spring of 2022.

**Conclusions:**

Developing RESOME has the potential to contribute to improved reporting, and such guidelines will make it easier to assess the effectiveness of social media interventions. Future work will be needed to evaluate our guidelines’ usefulness and practicality.

**International Registered Report Identifier (IRRID):**

PRR1-10.2196/31739

## Introduction

Social media use is becoming increasingly common; social media has been estimated to be used by more than half of the world’s population [1,2]. Given social media’s potential for promoting widespread public engagement, it is commonly incorporated into public health interventions [3,4]. The term *intervention* in this protocol refers to a spectrum of activities that range from national awareness-raising public health campaigns, such as awareness months, to more targeted activities that use social media groups to deliver information or interventions to specific audiences [5,6].

Systematic reviews of public health interventions involving social media have shown that social media has been used for a wide range of health promotion activities, such as weight management improvement, physical activity, smoking cessation, mental health improvement, the promotion of the use of health care services, HIV prevention, cancer prevention, and vaccination uptake [7-18]. These reviews highlight several potential benefits of social media interventions; they are generally found to be acceptable and feasible, and they offer unique advantages over traditional health messaging, such as the functionality of targeting information regarding specific populations, particularly those who may be hesitant to access in-person services [10,14,19,20].

Several methodological challenges however have been identified that hinder the ability to draw strong conclusions about the effectiveness of social media interventions. For example, some studies do not sufficiently describe interventions to allow for replicability, to understand the “active ingredients” of an intervention, or to understand the behavior change techniques used [11,13,15,16]. Very few studies explore the impacts on behavior change or quantitative clinical outcomes, and the majority instead focus on descriptions of engagement within a social media platform, such as views or shares, which may not indicate a genuine increase in knowledge or change in behavior [8,10,11,15,17]. Further, 3 systematic reviews reported that the majority of their included studies had a high or unclear risk of bias, which prevented a meta-analysis from being performed [8,13,21]. Many campaigns are not formally evaluated, and reports on social media campaigns are often not publicly available. These issues further contribute to the challenge of synthesizing research evidence. Furthermore, although social media interventions have been carried out in a variety of different populations, there are very few studies that actively explore and report the impact of social media interventions on health inequalities [7,11,22].

There are existing reporting guidelines that have some relevance to social media interventions, such as Criteria for Reporting the Development and Evaluation of Complex Interventions in Healthcare: Revised Guideline (CReDECI 2) for complex health care interventions, CONSORT (Consolidated Standards of Reporting Trials) for randomized controlled trials, Guidance for Reporting Intervention Development Studies in Health Research (GUIDED) for intervention development, and guidelines for using the theory of change to design and evaluate public health interventions and for better reporting on interventions (eg, Template for Intervention Description and Replication [TIDieR]) [23-27]. However, there are currently no reporting guidelines available that take into account the diverse and unique aspects of research on social media interventions. For example, not all evaluations of social media interventions have the same study design; it is not always feasible or possible to randomize participants in a trial, and some studies are observational in nature (eg, an evaluation of a national awareness campaign delivered by a third party) [19,20,28].

There is therefore a clear need for guidance to be developed on the reporting of social media interventions. The development of reporting guidelines that incorporate elements from existing and widely used behavior change theories and social media engagement frameworks could foster more robust evaluations that capture outcomes that have an impact on behavior change and engagement and inform policy decisions [19,29,30]. Specific guidelines for social media research could include additional domains about the methods for collecting data, provide technical information about social media platforms and the social context in which the research takes place, and address the ethical and privacy issues that are unique to social media research.

The aim of this project is to develop and establish consensus on which items should be included in reporting guidelines for social media interventions. The development of these guidelines will be achieved by conducting a series of Delphi surveys to achieve consensus on which items should be included.

## Methods

The methods that will be used in this project have been informed by guidance on the development of reporting guidelines that was produced by Moher et al [31] in 2010 and the EQUATOR (Enhancing the Quality and Transparency of Health Research) Network [32]. The development of our Reporting Guidelines for Social Media Research (RESOME) has been registered on the EQUATOR Network website [33].

### Design

**Figure 1:**
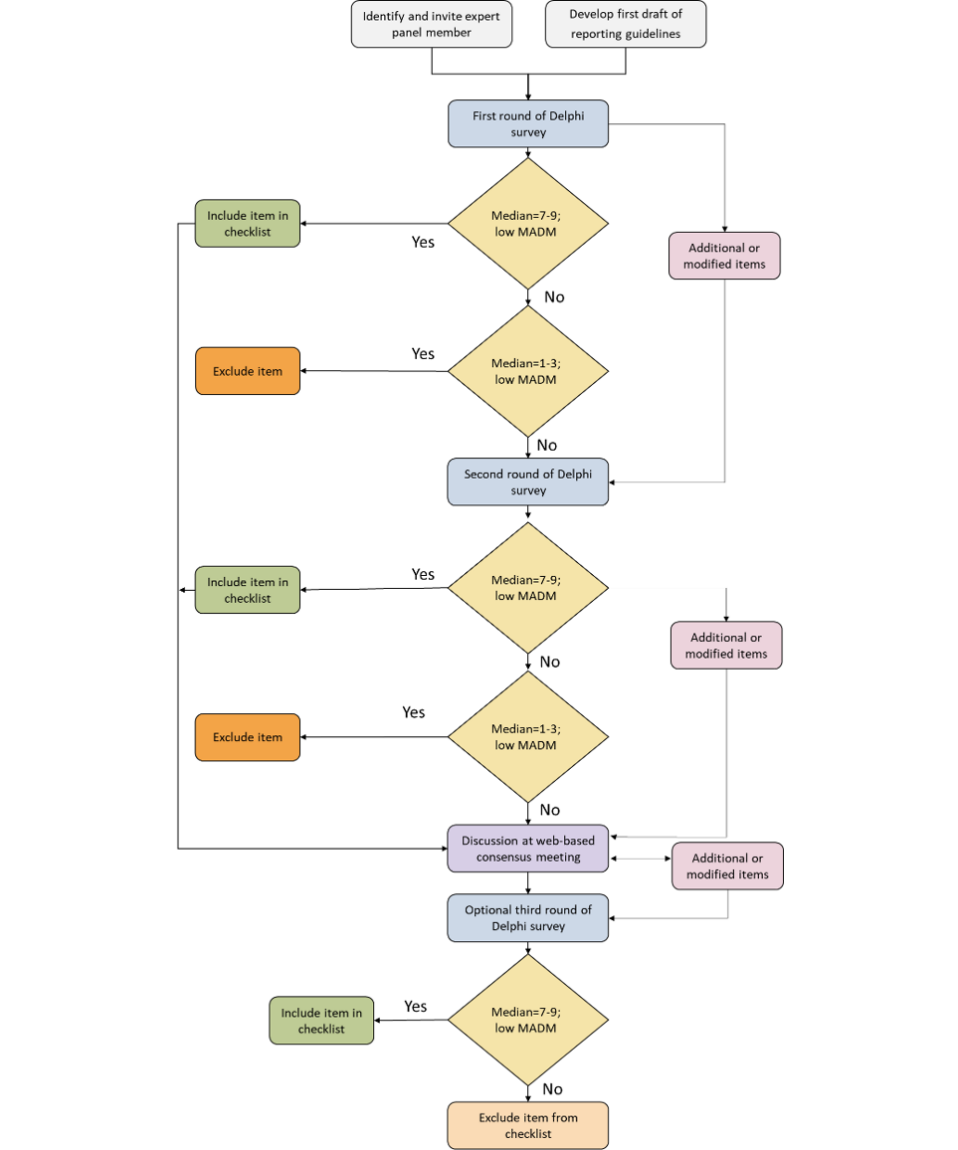
Delphi process flowchart. MADM: mean absolute deviation from the median.

The reporting guidelines will be developed by conducting a Delphi exercise, during which several rounds of questionnaires will be sent to experts and stakeholders. The questionnaires will ask them to rate their agreement with a series of statements until a level of consensus is reached. This will be followed by a web-based consensus meeting to finalize the reporting guidelines [34,35]. Figure 1 summarizes the processes of the Delphi method.

The project is led by an expert, multidisciplinary executive panel composed of 9 people from the fields of behavioral science, epidemiology, health services research, public health, social media, and statistics and patient and public representatives. The panel is responsible for providing academic oversight for the project and directly participating in some aspects of the research activities, such as identifying participants, chairing discussions, and contributing to developing research reports.

### Participants

As recommended by the EQUATOR Network [32] and the guidance produced by Moher et al [31] in 2010, stakeholders with expertise in statistics, epidemiology, and research methodology; primary care and public health professionals; journal editors; and patient and public representatives will be invited to form an expert panel in addition to researchers with experience in conducting or evaluating social media interventions. Potential participants will be identified via research networks and invited to take part by email. We will contact social media researchers and journal editors directly and advertise the project on social media. The invitation email will contain background information on the project; a link to the preprint of the protocol; a participant information sheet; and a privacy notice stating that any personal data will be collected, stored, and deleted in accordance with the UK General Data Protection Regulation (2018) [36]. Potential participants will be asked to share information about the project with colleagues and among their own research networks to increase the pool of potential participants. The final participant panel will include a maximum of 30 people. We will aim to ensure that the panel is diverse with respect to age, gender, ethnicity, and subject specialty.

### Delphi Survey

The purpose of the Delphi exercise is to generate a list of items for consideration at the consensus meeting [34]. The first iteration of the checklist items was developed based on previously conducted research and relevant guidelines. The current items are outlined in Table 1 [4,11,23-25]. Using questionnaires will allow for anonymous responses, which can help with avoiding influence from other members, and allow people from geographically dispersed countries to participate. The purpose of the web-based consensus meeting is to provide an opportunity for clarifying and discussing items.

Participants will be asked to rate their agreement with including each item on a scale of 1 to 9 (1=strongly disagree; 9=strongly agree). Each question will be accompanied by a free-text box, in which participants will be able to add comments, such as justifications for their responses, suggestions for alternative phrasing, or additional items.

After the first round, items for which there is consensus for inclusion will be retained (see *Statistical Analysis* section for further details). Each participant will receive the aggregate results of the survey, along with their own answers to the questions. Any items for which consensus is not reached will be included in a second round of surveys, along with any additional items or modifications identified through free-text responses. Participants will then be presented with the results of the first round and asked to rate the items again before the web-based consensus meeting.

**Table 1:** The draft of Reporting Guidelines for Social Media Research to be included in round 1 of the Delphi exercise.

Sections and item numbers		Description^a^
**Title and abstract**		
	1A	Indicate the use of social media in the title or abstract
	1B	Indicate the social media platform(s) in the title or abstract
**Methods: study design**		
	2A	Specify study design e.g., observational, experimental, qualitative
	2B	Specify if this study reports an evaluation of an existing social media campaign (e.g., a process evaluation) or a novel intervention
	2C	Specify the trial registration details and/or previously published protocol, if applicable
**Methods: participants and data**		
	3A	Describe the eligibility criteria for participants to be involved in the study or the population targeted by the intervention
	3B	Describe any methods used to identify participants
	3C	Describe how participants were recruited into the study
	3D	Explain if the data are publicly available and where it can be accessed
	3E	Describe the methods used to collect data e.g., search terms, social media metrics or data mining
**Methods: setting**		
	4A	Provide a summary of the social media platform(s), including the main features and how it is used
	4B	Provide information about the number of users of the social media platform(s) and their characteristics
	4C	Describe any relevant algorithms employed by the social media platform(s) at the time of the research which may have an impact on the way that information is shared and viewed
	4D	Provide details of any social, political and economic contextual factors relevant to the project e.g., ongoing public debates or events
	4E	Provide dates of exposure, follow-up, and data collection
**Methods: intervention**		
	5A	Describe the primary and secondary aims of the intervention or campaign (e.g., change in knowledge, awareness, intentions, behaviours, or health outcomes)
	5B	Describe how the social media intervention or campaign works with reference to behaviour change techniques, mechanisms of action, and targeted behaviour change
	5C	State whether the intervention or campaign was paid for or not
	5D	Describe the purchase model if used for any advertisements or promoted posts
	5E	Describe the implementation strategy for the social media campaign and any incentives for participants
	5F	Describe the resources required for the campaign or intervention e.g., time, money, technology, equipment, and partners
	5G	Provide a description of the intervention with sufficient detail to allow replication, including any links to audio/visual materials
	5H	Specify whether the intervention materials are subject to a copyright license or can be reused e.g., through a public or creative common license
	5I	Describe the creative process underlying the development of intervention materials, including an acknowledgement of any outside agencies involved in developing creative materials
	5J	Describe any aspects of the intervention which took place outside of social media e.g., television, print and radio communications
	5K	Describe any secondary features of the intervention or campaign which may impact the outcome measures e.g., sponsors, collaborators and links to other websites
**Methods: measures**		
	6A	Reach: Describe the demographic data of included participants, if applicable
	6B	Low-medium level engagement: Describe which measures were used to measure engagement with the intervention e.g., likes, comments, reposts
	6C	High-level engagement: Describe any measures for change in knowledge, awareness of offline activities e.g., measures of behaviour change, intentions, or health-related outcomes.
**Methods: ethics**		
	7A	Provide details of any ethical implications related to consent, privacy issues and data protection
	7B	Provide details of any safeguarding measures e.g., moderation of discussion
	7C	Describe any commercial interests related to the project e.g., funding, sponsorship, advertisements, endorsements
	7D	Describe how any commercial interests were communicated to participants
**Results: participant flow diagram**		
	8	Include a flow-diagram showing the number of people who were targeted, exposed to the intervention, engaged with the intervention, and included in the analysis, if applicable
**Results: participant characteristics**		
	9	Include a table showing demographic information for participation (by group if a control group is used), if applicable
**Results: outcomes**		
	10A	Report descriptive statistics of social media metrics, such as reach, views, likes and reposts
	10B	Include a description of novel content such a conversations, videos, or images
	10C	For measures of high-level engagement, provide an estimation of differences between groups or over time with a measure of precision (such as 95% confidence interval).
**Discussion: summary of results**		
	11	Describe the main results in the context of the objectives described in the methods section and any previously published research protocols or trial registrations
**Discussion: feasibility**		
	12	Discuss the feasibility of the campaign and intervention for wider implementation with reference to the benefits in relation to the resources required
**Discussion: generalizability**		
	13	Discuss which components are generalisable to other social media platforms and contexts e.g., for different target behaviours, socio-demographic groups or countries

^a^All descriptions appear exactly as they did when they were presented to participants.

### Consensus Meeting

In-person meetings are one of the major challenges of consensus meetings due to the cost and practicality of convening an international group of experts. Due to the ongoing COVID-19 pandemic, these meetings will be conducted on the internet, which will eliminate the cost of international travel and allow for the greater participation of experts in different countries.

The consensus meeting will be limited to 30 people to allow for effective communication and participation and will take place over 1 day. Prior to the meeting, preparations will be made, including developing the agenda and presentations relevant to the session; making sure that participants have information about joining the session; sharing the results of the Delphi exercise; and sharing any other relevant materials, such as the current item list. The meeting will be conducted by using web-based conferencing software, such as Zoom (Zoom Video Communications Inc) or Remo (Remo.co), and conversations will be recorded for future reference.

The consensus meeting will include presentations on the research background and the results of the Delphi exercise. Each item will be discussed in turn to allow participants to express their viewpoints in more detail and to ultimately reach consensus on items’ inclusion or exclusion in the reporting guidelines. Due to the meeting taking place on the internet, it will be possible to incorporate live and anonymous voting on items to break deadlocks. The meeting will also be used to discuss additional reporting guidelines, such as the inclusion of a participant flowchart or a summary of social media metrics; the name of the reporting guidelines; the publication and dissemination strategy; and the accompanying explanation and elaboration document.

### Statistical Analysis

For each item, the mean, SD, median, IQR, and mean absolute deviation from the median (MADM) will be used to assess the strength and extent of agreement [37]. Medians of 7 to 9 will be defined as strong support, medians of 4 to 6 will be defined as moderate support, and medians of 1 to 3 will be defined as no support. The MADM will be categorized as high (>2.68), medium (1.34-2.67), or low (<1.33), with a low MADM representing a high-level consensus. Items will be included if the median score is ≥7 and the MADM is low (<1.33). Items will be excluded if the median score is ≤3 and the MADM is low (<1.33). Items that do not meet these criteria after the second round will be discussed in the web-based consensus meeting, and an additional, third Delphi round will be conducted after the meeting. If consensus is not reached by this stage, a decision will made by the executive panel.

Free-text comments from each Delphi survey will be qualitatively analyzed by using a thematic analysis [38]. The main themes will be reported to the executive panel and the participants in order to identify any additional items or item modifications that need to be included in the next round of the survey.

### Developing the Reporting Guidelines and an Explanatory Document

After the consensus meeting, the reporting guidelines will be published in the form of a paper outlining the need for the new guidelines and how the guidelines were developed, along with the finalized checklist for reporting. Prior to publication, guidelines will be piloted to check for understanding and to simplify the language, if necessary.

An explanation and elaboration document will be published alongside the reporting guidelines. The document will explain why each reporting guideline is important and provide examples for each item.

### Ethics Approval

Ethical approval was obtained in June 2021 from the University College London ethics committee (ID number: 14687/004) to contact participants and to collect and store their responses in accordance with the UK General Data Protection Regulation (2018). All participants will complete a web-based consent form prior to taking part in the study and have the right to withdraw themselves from the study.

### Dissemination

After the publication of the reporting guidelines, a range of strategies will be implemented to improve the usability and implementation of the reporting guidelines (Textbox 1).

**Strategies for improving the usability and implementation of Reporting Guidelines for Social Media Research.**

**Strategies**

- Ensuring that the guidelines are freely available and are in an editable format
- Creating a dedicated website containing additional resources
- Recording the guidelines on the EQUATOR (Enhancing the Quality and Transparency of Health Research) Network database
- Obtaining journal endorsement and endorsement from other organizations
- Presenting the guidelines at relevant conferences and meetings
- Writing blog posts and other social media posts about the guidelines
- Developing and delivering web-based training in using the guidelines
- Translating the guidelines into different languages

## Results

As of January 2021, the executive panel has been convened and has drafted the first version of the RESOME checklist. The recruitment of participants and round 1 of the Delphi survey took place between July and December 2021. Round 2 is due to take place in February 2022, and the web-based consensus meeting will be scheduled for the spring of 2022. The finalized guidance and supporting explanatory document will be published by August 2022.

## Discussion

### Study Implications

Social media interventions are a relatively recent phenomenon that has attracted a high level of research interest; however, there is a clear need for reporting, as evidenced by the large number of reviews that report a lack of robust and rigorously conducted studies [8,10,11,13,15-17,21]. The rapid changes in the way that people use social media make investigating methods for promoting the public’s engagement with health messages via social media a high priority. The development of and adherence to reporting guidelines for social media interventions will facilitate replicability and allow for a better understanding of which interventions are effective and for whom they are effective.

Delphi surveys are a validated consensus method that involve using anonymous surveys to quantify levels of consensus according to an a priori statistical analysis plan. This approach is especially beneficial because it allows for the equal contribution of participants and prevents dominating personalities from impacting decision-making [39]. Qualitative and quantitative data will be analyzed to achieve a deeper understanding of participants’ responses. The main limitation of Delphi surveys is the lack of opportunities for nuanced discussions or clarifications among individuals. This limitation will be addressed with an interactive and deliberative consensus meeting. Due to the web-based nature of the consensus building process, a broader range of stakeholders than what would normally be possible will be recruited. By including participants from different geographical regions who would not normally be able to participate, it is possible to achieve a global perspective and an increased level of diversity in terms of participants [40]. The publication of this protocol provides an opportunity to improve the research methods (via feedback from reviewers) and transparency for the process of including and excluding items.

### Limitations

There are some limitations to this project that must be acknowledged. First, the recruitment of the expert panel may be challenging in terms of finding and engaging people with relevant expertise and diverse opinions. Second, the reporting guidelines that will be produced in this project may not be applicable or relevant to all types of social media interventions. It is expected that these social media reporting guidelines could and should be used by researchers in conjunction with other existing reporting guidelines (eg, CReDECI 2, CONSORT, GUIDED, and TIDieR) [23-27]. Third, the field of social media research is fast-moving, and as such, any reporting guidelines will require regular reviews and updates to reflect changes in methodological approaches. Finally, once the reporting guidelines have been published, several steps will be taken to disseminate and encourage their use. However, this will require engagement and endorsement by relevant journals and third-party organizations.

### Conclusions

There is a lack of high-quality research on social media interventions, which limits our understanding of their effectiveness. This protocol outlines the methods that will be used to develop reporting guidelines for social media interventions. The development of these guidelines are a first step toward improving the quality, rigor, and robustness of social media interventions. Future work will be needed to evaluate the guidelines’ usefulness and practicality.

## Abbreviations

CONSORT: Consolidated Standards of Reporting Trials
CReDECI 2: Criteria for Reporting the Development and Evaluation of Complex Interventions in Healthcare: Revised Guideline
EQUATOR: Enhancing the Quality and Transparency of Health Research
GUIDED: Guidance for Reporting Intervention Development Studies in Health Research
MADM: mean absolute deviation from the median
NIHR: National Institute for Health Research
RESOME: Reporting Guidelines for Social Media Research
TIDieR: Template for Intervention Description and Replication
